# Pharmacological inhibition of MDM4 alleviates pulmonary fibrosis

**DOI:** 10.7150/thno.81993

**Published:** 2023-05-08

**Authors:** Qianru Mei, Zhenhua Yang, Zhengkai Xiang, He Zuo, Zijing Zhou, Xiaochuan Dong, Ludan Zhang, Wenhui Song, Yi Wang, Qinghua Hu, Yong Zhou, Jing Qu

**Affiliations:** 1 School of Basic Medicine, Tongji Medical College, Huazhong University of Science and Technology, Wuhan 430030, China.; 2 Department of Thoracic Surgery, Hubei Cancer Hospital, Tongji Medical College, Huazhong University of Science and Technology, Wuhan 430079, China.; 3 Department of Pulmonary and Critical Care Medicine, Second Xiangya Hospital, Central South University, Changsha, Hunan 410011, China.; 4 Department of Pathology, Union Hospital, Tongji Medical College, Huazhong University of Science and Technology, Wuhan 430022, China.; 5 Division of Pulmonary, Allergy, and Critical Care Medicine, Department of Medicine, the University of Alabama at Birmingham, Birmingham, AL, United States.

**Keywords:** Idiopathic pulmonary fibrosis, MDM4-p53 pathway, MDM4 inhibitor, XI-011, Therapeutic drug.

## Abstract

Idiopathic pulmonary fibrosis (IPF) is a progressive and fatal lung disease of unknown etiology with no cure. A better understanding of the disease processes and identification of druggable targets will benefit the development of effective therapies for IPF. We previously reported that MDM4 promoted lung fibrosis through the MDM4-p53-dependent pathway. However, it remained unclear whether targeting this pathway would have any therapeutic potential. In this study, we evaluated the efficacy of XI-011, a small molecular inhibitor of MDM4, for treating lung fibrosis. We found that XI-011 significantly reduced MDM4 expression and increased the expression of total and acetylated p53 in primary human myofibroblasts and a murine fibrotic model. XI-011 treatment resulted in the resolution of lung fibrosis in mice with no notable impact on normal fibroblast death or the morphology of healthy lungs. Based on these findings, we propose that XI-011 might be a promising therapeutic drug candidate for treating pulmonary fibrosis.

## Introduction

Human idiopathic pulmonary fibrosis (IPF) is a chronic and progressive lung disease associated with poor prognosis and significant morbidity 1. Although two US FDA-approved drugs, nintedanib and pirfenidone, are available for treating IPF, they only slow down the disease progression 2, 3, but cannot cure the disease. Additionally, severe side effects are observed in a subgroup of patients 4. There is an urgent need to develop new and effective medications for the devastating fibrotic lung disease.

The etiology of IPF remains unclear. It is thought that IPF occurs following repetitive lung injury 5. Epithelial lung injury activates lung healing and regeneration, whereas IPF may result from aberrant repair and regeneration following lung injury 6-8. p53 is an established tumor suppressor which induces cell cycle arrest, apoptosis, senescence, and innate immunity 9. p53 expression was absent or negligible in IPF lungs compared with normal subjects 10, suggesting that its dysfunction may negatively affect IPF formation.

MDM2 and MDM4 proteins, deregulated in many human cancers, exert their oncogenic activity predominantly by inhibiting the p53 tumor suppressor 11. We recently demonstrated that reduced Mdm4 expression or its genetic ablation promoted lung fibrosis resolution by activating the Mdm4-p53 pathway in aged mice, highlighting the potential of MDM4 as a therapeutic target for the treatment of lung fibrosis 12.

In this study, we examined the therapeutic potential of a commercially available small-molecule p53 activator, NSC146109 (XI-011), in lung fibrosis. *In vitro* studies showed that XI-011 inhibited MDM4 expression and increased total and acetylated p53 levels. The ability of XI-011 to reverse lung fibrosis was verified in a bleomycin injury mouse model. XI-011 had little effect on the morphology of normal mouse lungs. Mechanistic studies demonstrated that XI-011 exerted its anti-fibrotic properties by sensitizing lung myofibroblasts to apoptosis and promoting the clearance of myofibroblasts by macrophages. These findings suggest that XI-011 is a promising drug candidate for treating pulmonary fibrosis.

## Methods

### Antibodies and inhibitors

Anti-MDM4 and anti-CD68 antibodies were obtained from ThermoFisher Scientific. Anti-acetylated p53, anti-p53, anti-fibronectin, anti-Collagen I, and anti-αSMA antibodies were purchased from Cell Signaling Technology (Danvers, MA, USA). Anti-DD1α and anti-Fas antibodies were purchased from Abcam (Cambridge, MA, USA). The anti-GAPDH antibody was from Abclonal (Wuhan, China). NSC146109 (XI-011) was purchased from Sigma-Aldrich. SJ172550 was obtained from MedChem Express (Monmouth Junction, NJ, USA).

### Human tissues

The studies involving human subjects were approved by Medical Ethics Committee at the Huazhong University of Science and Technology. Lung tissue biopsies were obtained from three IPF patients (all males, age > 60 y), who underwent thoracic surgery at Hubei Cancer Hospital of Huazhong University of Science and Technology. Three normal lung biopsies from patients undergoing non-small cell lung cancer resection (incisal edge > 5 cm) (two males, one female, age > 45 y) were used as controls.

### XI-011 treatment of myofibroblasts and CCK-8 assay

Fibroblasts were plated at 4×10^4^ cells/well in a 6-well plate with varying concentrations of XI-011 and incubated at different time points as indicated in the text. Cells were then harvested by scraping for protein and mRNA detection.

Cell viability and cytotoxicity were evaluated using a standard CCK-8 reagent assay (Biosharp, Hefei, China). Briefly, human and mouse fibroblasts were plated at a density of 1 × 10^4^ cells/well in 96-well plates and treated with XI-011 as indicated. The cells were then treated with 10 μl of CCK-8 reagent and incubated for 4 h. The absorbance was read at 450 nm using a microplate reader (ThermoFisher Scientific).

### Animals and experimental protocol

Six- to eight-week-old pathogen-free female mice were used in this study (n=8-10 per group). Bleomycin sulfate (Sigma) was dissolved in 50 μl sterile saline solution and intratracheally introduced into mice as a single dose of 3 mg/kg body weight. Control mice received 50 μl saline. For the XI-011 treatment, mice were given a dosage of 1 mM/kg body weight (50 μl) or an equal volume of saline every other day by intratracheal inhalation 10 times in total. The lung tissues were extracted for experimental analysis 1-2 days after the last administration of the inhibitor.

### Real-time quantitative PCR assay

Total RNA was extracted from either cell lysate or the snap-frozen lungs of mice with TRIzol reagent (Takara) as described 13. Briefly, 1 µg total RNA was reversely transcribed into cDNA with a HiScript RT SuperMix (Takara). Quantitative PCR (qPCR) reactions were performed using qPCR SYBR Green Master Mix (Vazyme, Nanjing, China) according to the manufacturer's instructions. Each sample was run in triplicate. Relative quantification was calculated using the comparative CT method. Delta CT values of the target gene were normalized to GAPDH.

### Western blotting assay

Western blotting (WB) was conducted as described 14, 15. Briefly, cells were directly lysed in RIPA buffer (Sigma) supplemented with protease inhibitors (Sigma). For lung tissue, the samples were resuspended in RIPA lysate buffer and ground well using a grinding rod. Samples were ultrasonicated several times and then centrifuged at 12,000 rpm for 20 minutes to obtain clear lysates for subsequent protein studies. Cell lysates containing 20-40 µg total proteins were loaded onto SDS-PAGE gel under reducing conditions. A dilution of 1:1000 was used for primary all antibodies except GAPDH-HRP, which was diluted at 1:10000. SuperSignal West Pico PLUS substrate (ThermoFisher Scientific) was added to visualize the target bands using a ChemiDoc imaging system. Images were scanned and bands were quantified by ImageJ 16. Densitometry values of the target protein were normalized to GAPDH and subjected to statistical analysis.

### Lung histology, IHC, and IF confocal microscopy

The lung tissue was fixed with 4% (w/v) paraformaldehyde solution overnight and then embedded with paraffin. The paraffin was then cut into 5 μm lung tissue sections to assess interstitial lung injury with hematoxylin and red (H&E) staining, and collagen deposition with Masson tricolor (collagen stained blue). Masson's trichrome staining for collagen and H&E staining were performed according to the manufacturer's instruction (Solarbio, Beijing, China).

For IHC, dewaxed lung tissue sections were immersed in 10 M sodium citrate buffer (pH 6.0) and heated for 30 min to unmask the antigen. The immunohistochemistry kit (Zsbio, Beijing, China) was used according to the manufacturer's recommendations. The nuclei were stained with hematoxylin after coloring with DAB reagent. The fully automated slice scanning system was used to detect the pathological changes and protein expression in lung tissue slices. Semi-quantitative analysis of IHC images was performed using ImageJ 16.

For immunofluorescence (IF) analysis, freshly collected lung tissue was buried in OCT and frozen at -80 ºC for long-term storage, and then the frozen OCT tissue was cut into 10 μm lung tissue sections for immunofluorescence staining. The tissue sections were then blocked with 5% normal goat serum and stained with primary antibodies diluted in PBS containing 1% goat serum and 0.3% Triton X-100. Subsequent visualization was performed after incubation with Alexa Fluor 488 and 596-conjugated secondary antibodies (ThermoFisher Scientific). Nuclei were stained with DAPI (ThermoFisher Scientific). Images were captured with a fluorescence microscope. The distribution and number of immunopositive stained cells or the immunopositive areas were identified and quantified as the relative stained areas (%) using imaging software (ZEISS).

### *In vitro* bleomycin treatment and TUNEL assay

Fibroblasts were treated with 2 μg/ml of bleomycin or an equal volume of vehicle for 24 h. The supernatant was then replaced with fresh medium containing 1μM XI-011. Indicated assays were performed at different time points post-incubation.

TUNEL staining was performed using a kit (Abbkine, Wuhan, China) according to the manufacturer's instructions. In conjunction with IF staining, the TUNEL assay was performed, followed by incubation with primary antibodies for 24 h. Fluorochrome-conjugated secondary antibodies were used according to the manufacturer's recommendation. Finally, nuclei were stained with DAPI.

### PA hydrogels preparation and Hydroxyproline (Hyp) content assay

The preparation of PA hydrogels and Hyp content assay were described in our previous publications 12, 17.

### Statistical analysis

Experimental results were obtained from biological replicates averaged to generate a single summary value, recorded as mean ± SD under each condition. Two experimental groups were compared using the Student's t-test or the Student's t-test with Welch's correction for unpaired data. More than two groups were compared using one-way ANOVA with Bonferroni's correction. p<0.05 indicated statistical significance.

## Results

### MDM4 expression is associated with αSMA-positive myofibroblasts in both human IPF and bleomycin-induced experimental lung fibrosis in mice

**Figure 1:**
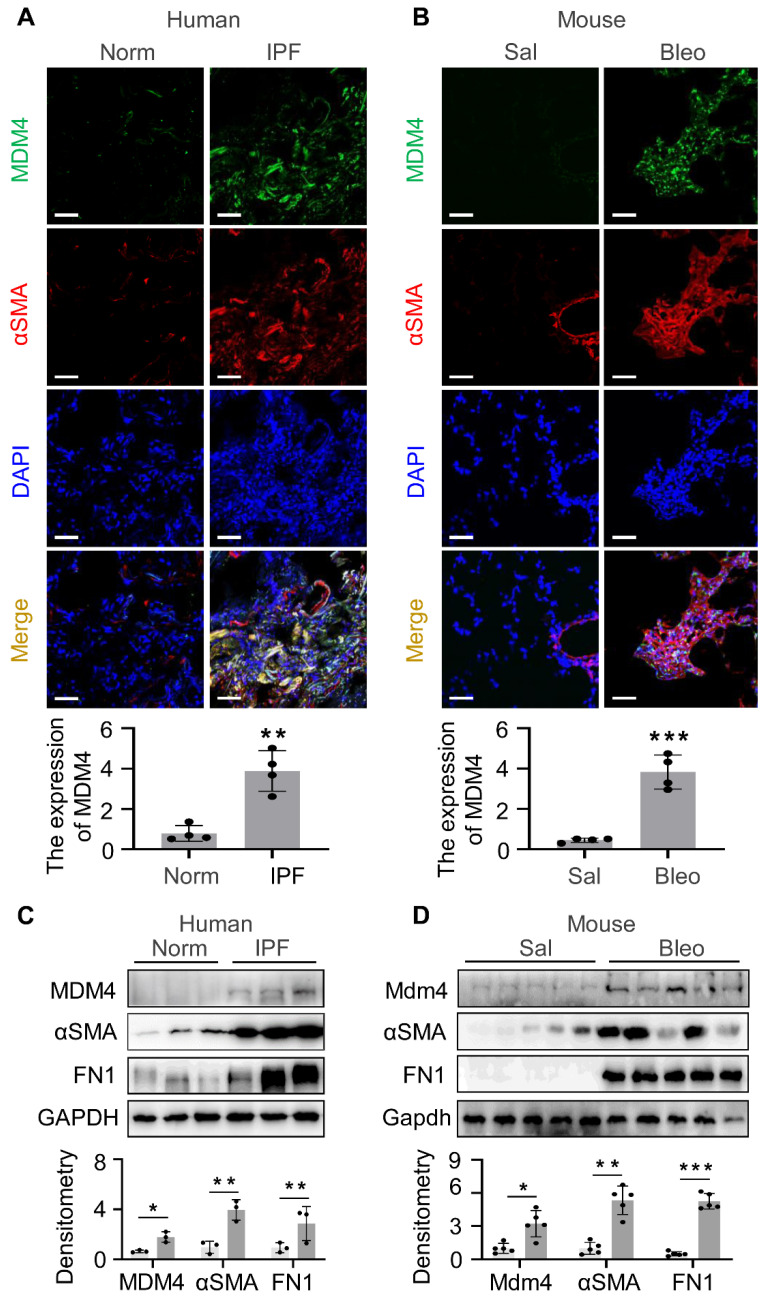
MDM4 is highly expressed in the fibrotic lesions of human IPF and bleomycin-induced mouse lung fibrosis. (A and B) Confocal IF microscopy to evaluate the expression of MDM4 and αSMA in the fibrotic lungs of IPF patients (A) and bleomycin-treated mice (B). The relative intensity of MDM4 signals was quantified for (A) and (B), respectively. (C) Immunoblotting analysis of the expression of MDM4, αSMA, and FN1 in primary lung myofibroblasts isolated from patients with IPF or normal control (Norm) subjects (n = 3 per group). (D) Immunoblotting analysis of Mdm4 expression in primary lung fibroblasts isolated from bleomycin-induced mice fibrotic lung tissues and control mice (n = 5 per group). Relative protein levels were quantified to GAPDH by ImageJ. Data were presented as the mean ± SD of three separated experiments. *, P < 0.05; **, P <0.01; ***, P <0.001 (ANOVA). Scale bars: 50 μm.

Increased expression of MDM4 in human and mouse fibrotic lungs has been described 12. We verified the enrichment of MDM4 in fibrotic lesions by immuno-fluorescent (IF) staining of optimal cutting temperature compound (OCT)-embedded lung tissues collected from human IPF patients and 2-month-old- mice subjected to intratracheal bleomycin or saline treatment. Lung fibrosis in human lung tissues was confirmed by Masson's trichrome staining (Figure S1). Consistent with our previous findings, a higher MDM4 signal was present in fibrotic lungs, whereas only a faint signal was detected in normal human or saline-treated mouse lung tissues (Figure 1A-B). We also observed the MDM4 signal localized in lung myofibroblasts positive for αSMA, a hallmark of profibrotic myofibroblasts, in human IPF (Figure 1A).

We further verified the expression of MDM4 in fibrotic tissues by isolating primary lung fibroblasts from IPF patients (Figure 1C) and bleomycin-induced mice (Figure 1D). The protein levels of MDM4, αSMA, and fibronectin 1 (FN1) were higher in fibrotic cells than in the control cells (Figure 1C-D). These results confirmed that MDM4 expression is associated with fibrotic lesions in the lung.

### XI-011 inhibits stiff matrix-dependent MDM4 expression

MDM4 has been reported to be a promising target in treating human IPF 12. In this study, we sought to determine whether pharmacological inhibition of MDM4 could alleviate lung fibrosis. We first tested the effects of two small molecular inhibitors of MDM4, SJ-172550 18, 19 and NSC149109 (XI-011) 20, on MDM4 expression and p53 activity. Immunoblotting analysis showed that the MDM4 protein level was dose-dependently reduced after treatment with XI-011 for 6 - 12 h in A549 cells (Figure S2A). The reduction of MDM4 was accompanied by a substantial increase in both the protein level and acetylation of p53. In contrast, MDM4 (and mRNA in mouse fibroblast), p53, and acetylated p53 levels were not affected by SJ-172550 treatment (Figure S2B), suggesting that SJ-172550 might function as an MDM4 inhibitor in a cell type-specific manner.

**Figure 2:**
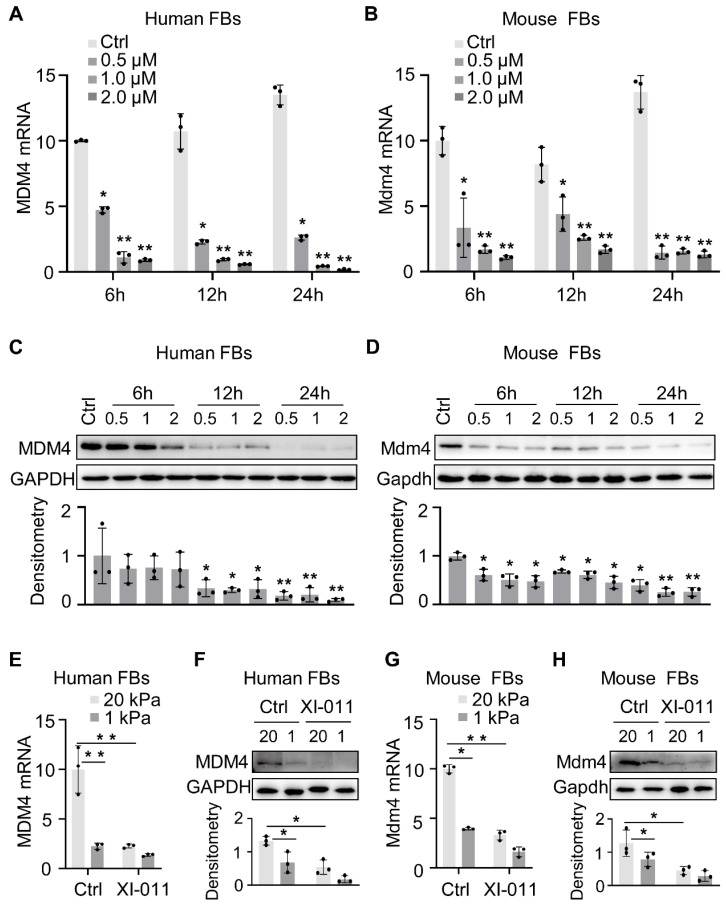
XI-011 inhibits MDM4 expression. Primary human and mouse lung fibroblasts (FBs) were isolated and co-cultured with varying doses of XI-011(A-D). The mRNA level of MDM4 (A and B) was determined by qPCR. Indicated proteins including MDM4, total p53, and acetylated p53 (Ac-p53) were detected by WB (C and D). Human or mouse FBs were co-cultured with 1 μM XI-011 on 1 or 20 kPa of polyacrylamide (PA) gels for 24 h (E-H). MDM4 expression was determined by qPCR (E and G) and WB (F and H), respectively. GAPDH was used as an internal reference control in qPCR analysis and as a loading control in WB analysis. Relative protein levels were quantified to GAPDH by ImageJ. Data were presented as the mean ± SD of three or four separate experiments. *, P < 0.05; **, P < 0.01 (ANOVA).

When primary lung fibroblasts were isolated and treated with XI-011, inhibition of the MDM4 mRNA expression was found to be dose-dependent manner (Figure 2A-B). Both human and mouse lung Fibroblasts showed an approximate two-fold reduction of MDM4 mRNA at 6 h post-incubation with XI-011 (Figure 2A-B). Immunoblotting confirmed a dramatic decrease in the MDM4 protein to a nearly undetected level at 24 h post-treatment (Figure 2C-D). These data indicated that XI-011 inhibited MDM4 expression at transcriptional and translational levels.

Matrix stiffness is a crucial mechanical factor that promotes lung fibrogenesis 21-23. We previously demonstrated that extracellular matrix stiffness increased MDM4 expression at mRNA and protein levels in lung fibroblasts 12. To test if XI-011 could inhibit stiff matrix-induced MDM4 expression, we cultured primary human and mouse lung fibroblasts on the polyacrylamide matrix substrate with stiffness comparable to normal and fibrotic lungs in the presence or absence of 1 μM of XI-011. In the vehicle-treated group, lung fibroblasts cultured on the stiff matrix (20 kPa) mimicking the fibrotic lungs expressed higher levels of MDM4 mRNA and protein than cells cultured on the soft matrix (1 kPa) mimicking normal lungs. In the XI-011-treated group, MDM4 expression was markedly reduced at mRNA (Figure 2E and 2G) and protein levels (Figure 2F and 2H) after 24 h. These results suggested that XI-011 inhibits MDM4 expression in response to matrix stiffening.

### XI-011 significantly induces lung myofibroblast apoptosis with little effect on normal cell viability

**Figure 3:**
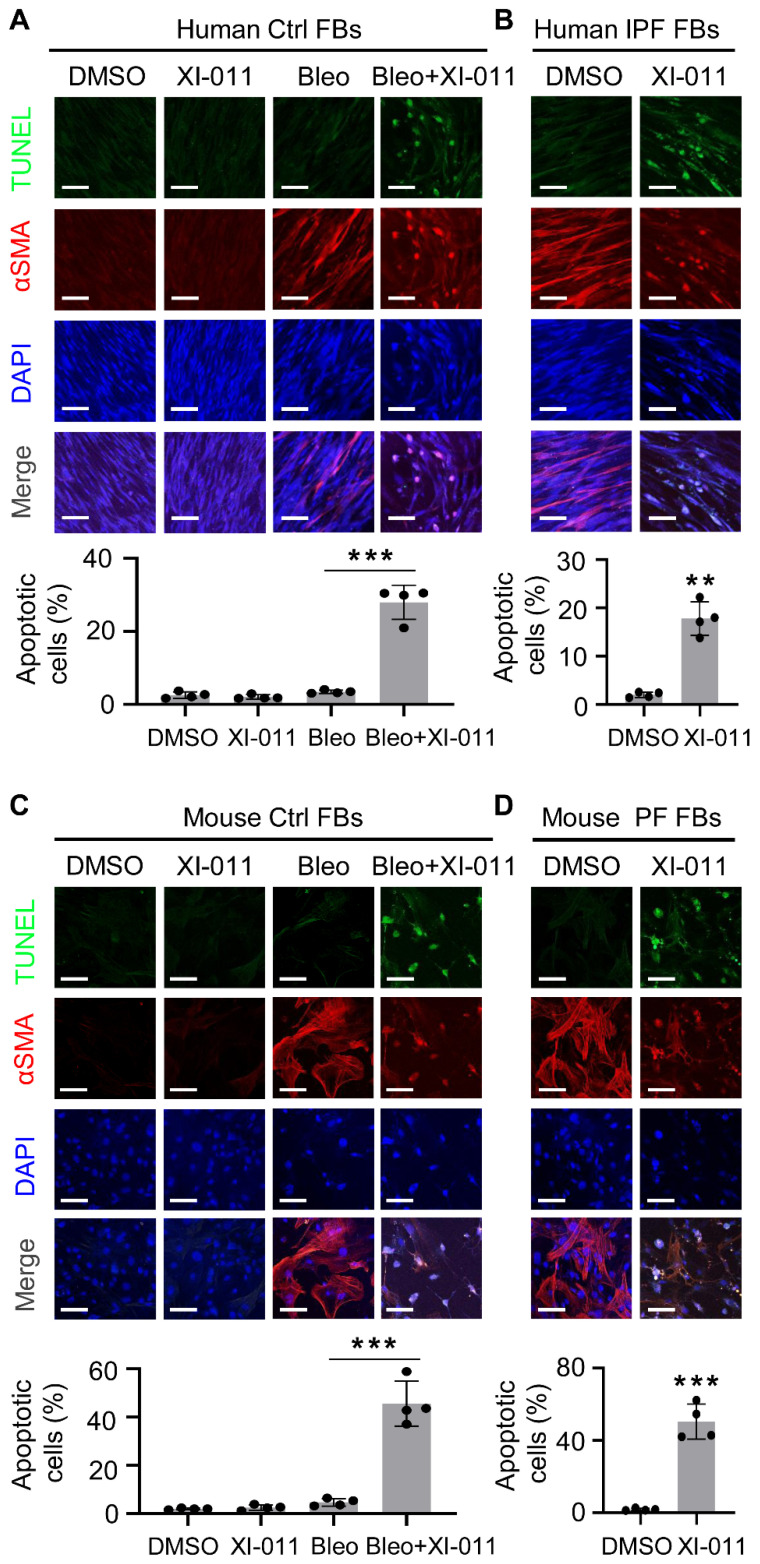
XI-011 induces apoptosis in lung myofibroblast *in vitro*. TUNEL and confocal IF microscopy were used to evaluate the effect of XI-011 on the apoptosis of bleomycin-induced myofibroblasts (A and C) or myofibroblasts derived from fibrotic lungs (B and D). Quantitative IF analysis was performed in 4 randomly selected areas. **, P < 0.01; ***, P <0.001 (ANOVA). Scale bars: 100 μm.

It has been reported that XI-011 induces apoptosis in several types of cancer cells 24-26. We isolated fibroblasts from the normal lung regions of patients with lung tumors and myofibroblasts from the fibrotic region of lungs from IPF patients to determine the effects of XI-011 on apoptosis *in vitro*. αSMA expression was employed to verify the differentiation of fibroblasts to myofibroblasts. We found that XI-011 had little effect on the viability of lung fibroblasts, whereas it induced substantial apoptosis of myofibroblasts (Figure 3A). Moreover, XI-011 significantly inhibited αSMA expression and caused cells to undergo apoptosis in IPF-derived myofibroblasts (Figure 3B). Similar results were observed with mouse lung myofibroblasts (Figure 3C-D). In addition, XI-011 more profoundly inhibited myofibroblast proliferation than fibroblast proliferation (Figure S3A-D). Whether XI-011 could induce injury in epithelial cells was tested by performing *in vitro* TUNEL and CCK8 assays on normal human bronchial epithelial cells (BEAS-2B) and murine lung epithelial cells (MLE12). We found that while bleomycin induced apoptosis in lung epithelial cells, the addition of XI-011 had little effect on cell apoptosis (Figure S4A-B). The CCK8 assay confirmed that XI-011 had minor effects on lung epithelial cell proliferation (Figure S4C-D). These findings indicated that XI-011 promotes lung myofibroblast apoptosis while it does not significantly affect the viability of normal lung cells.

### XI-011 promotes MDM4-p53-dependent Fas expression by lung myofibroblasts

**Figure 4:**
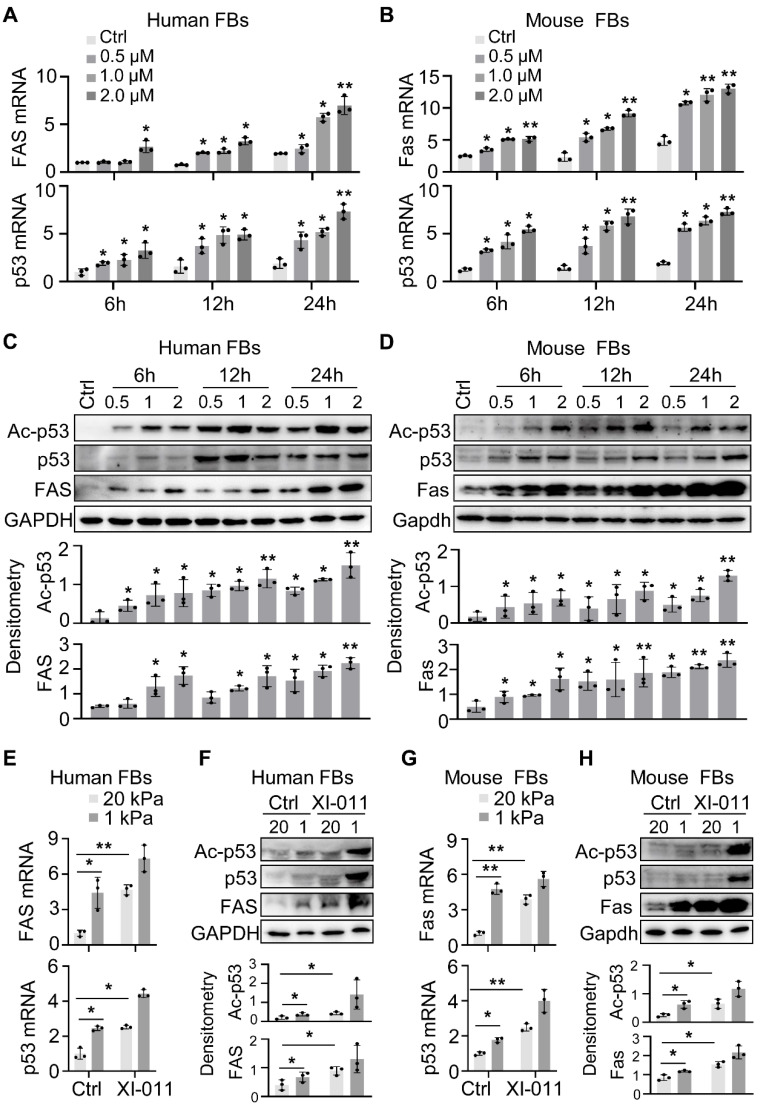
XI-011 promotes MDM4-p53-dependent FAS expression by lung fibroblasts. Primary human and mouse lung fibroblasts (FBs) were isolated and co-cultured with varying doses of XI-011 (A-D). Samples were collected at different time points and the expressions of indicated items were analyzed by qPCR (A and B) and WB (C and D), respectively. Human or mouse FBs were co-cultured with 1 μM XI-011 on 1 or 20 kPa of polyacrylamide (PA) gels for 24 h (E-H). The expressions of indicated items were analyzed by qPCR (E and G) and WB (F and H), respectively. GAPDH was used as an internal reference control in qPCR analysis and as a loading control in WB analysis. Relative protein levels were quantified to GAPDH by ImageJ. Data were presented as the mean ± SD of three separate experiments. *, P < 0.05; **, P < 0.01 (ANOVA).

MDM4 promotes cell proliferation by inhibiting the expression and transcriptional activity of p53 11, 27. We recently reported that reducing matrix stiffness increased the MDM4-p53-dependent expression of Fas, which played a role in lung myofibroblast apoptosis 12. We tested whether XI-011 could increase Fas expression by culturing primary human and mouse fibroblasts in the presence or absence of varying concentrations of XI-011 for 24 h. We found that XI-011 increased p53 and Fas expression at mRNA and protein levels in both human and mouse fibroblasts (Figure 4A-D) and also activated the acetylated p53 (Ac-p53) protein (Figure 4C-D). Further analyses confirmed that the soft matrix promoted the expression of Fas, p53, and Ac-p53 (Figure 4E-H). The addition of XI-011 greatly enhanced the expression of total p53 and Ac-p53, especially in the soft matrix (Figure 4F and 4H). These data suggested that XI-011 promotes the expression and activity of p53, possibly contributing to increased Fas expression in lung myofibroblasts.

### XI-011 increases the expression of DD1a and CX3CL1 in lung myofibroblasts

**Figure 5:**
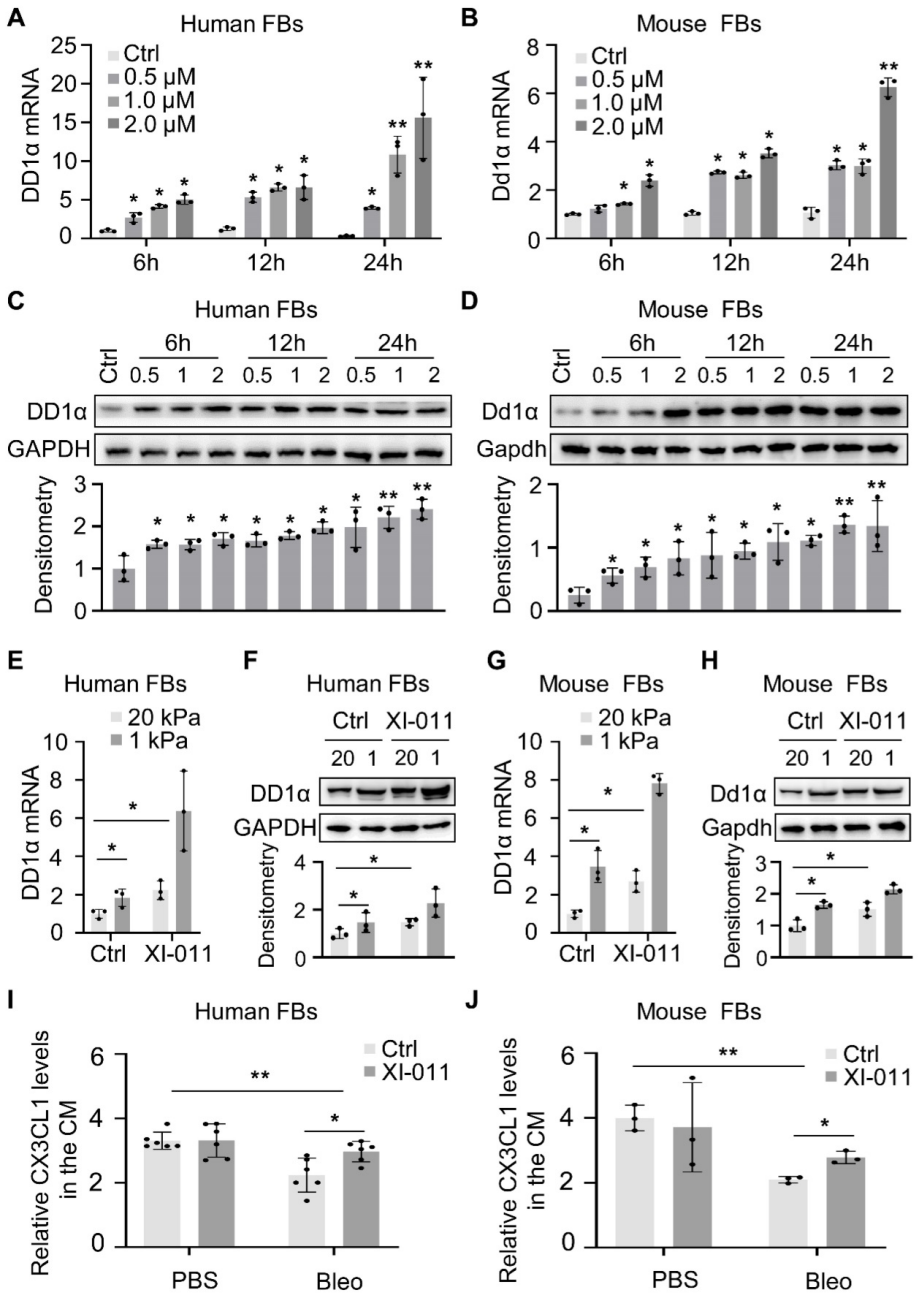
XI-011 promotes MDM4-dependent expression of DD1a, CX3CL1 by lung fibroblasts. Primary human and mouse lung fibroblasts (FBs) were isolated and co-cultured with varying doses of XI-011 (A-D). Cells were collected at different time points and the expression of DD1a was analyzed by qPCR (A and B) and WB (C and D), respectively. Human or mouse FBs were co-cultured with 1 μM XI-011 on 1 or 20 kPa of polyacrylamide (PA) gels for 24 h (E-H). The expression of DD1a was analyzed by qPCR (E and G) and WB (F and H), respectively. Levels of CX3CL1 in the conditioned medium (CM) were determined by ELISA (I and J). Relative protein levels were quantified to GAPDH by ImageJ. Data were presented as the mean ± SD of three separate experiments. *, P < 0.05; **, P < 0.01 (ANOVA).

The knockdown of MDM4 expression by siRNAs has been shown to facilitate the macrophage-mediated clearance of lung myofibroblasts by upregulating DD1α and CX3CL1 expression 12. In this study, *in vitro* experiments showed that XI-011 increased the expression of DD1α in human and mouse fibroblasts (Figure 5A-H). We collected the supernatant from bleomycin/saline-treated myofibroblasts (described in Figure 3 legend) and evaluated the effect of XI-011 on CX3CL1 expression. ELISA assay showed that the CX3CL1 level was slightly elevated (statistically significant) by XI-011 in human and mouse myofibroblasts (Figure 5I and 5J). In contrast, the expression of CX3CL1 was not affected by XI-011 in normal fibroblasts (Figure 5I and 5J). These results indicated that XI-011 promotes the expression of DD1a and CX3CL1 in myofibroblasts.

### Inhibition of MDM4 by XI-011 alleviates lung fibrosis

**Figure 6:**
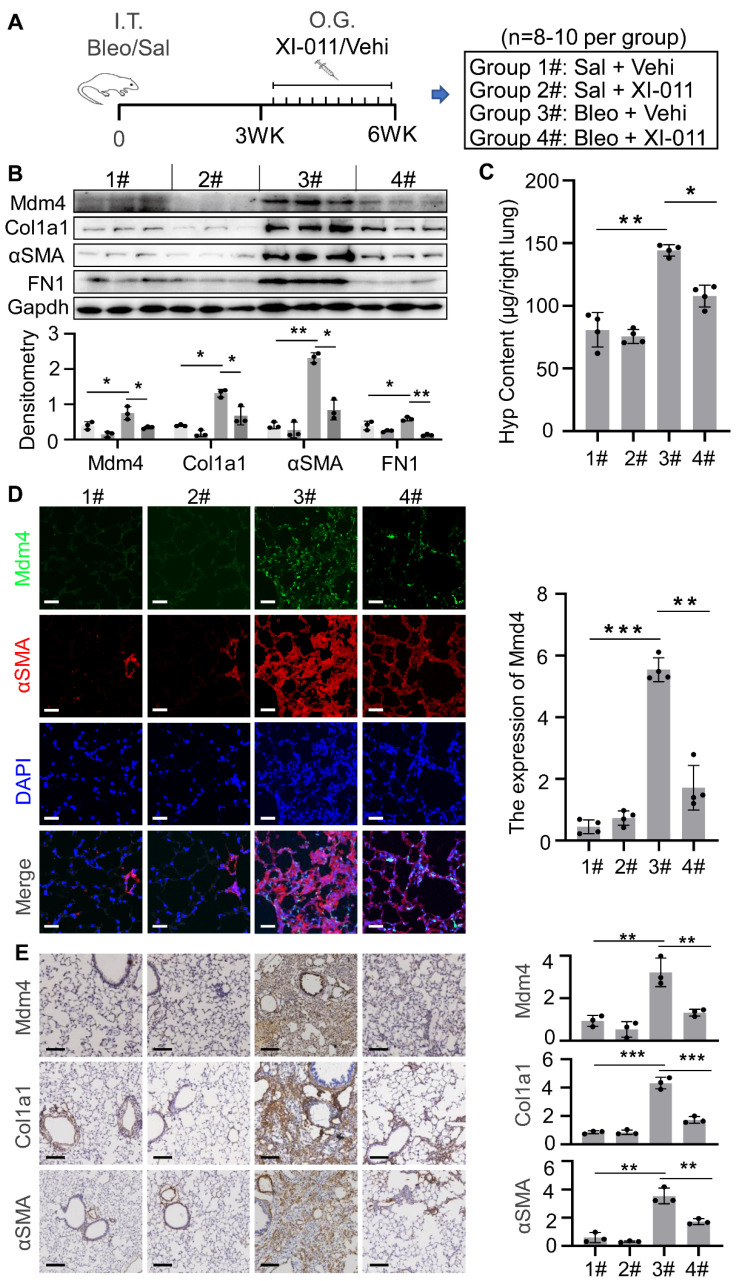
XI-011 promotes lung fibrosis resolution in mice. (A) Scheme for the animal experiments. I.T. Bleo/Vehi, intratracheal instillation of bleomycin or Vehicle (saline); O.G. XI-011/Vehi, intratracheal inhalation of XI-011 or Vehicle (DMSO). (B) Primary lung myofibroblasts were isolated from each group and protein levels of Mdm4, Ac-p53, total p53, Col1a1, αSMA, and FN1 were determined by WB. Gapdh was used as the loading control. Relative protein levels were quantified to Gapdh by ImageJ. Data were presented as the mean ± SD of three or four separate experiments. (C) The lung collagen deposition was quantified by Hyp assay (n = 4 per group). (D) Confocal IF staining to analyze the expression Mdm4 and αSMA. (E) IHC analysis of Mdm4, Col1a1, and αSMA expressions in lung tissues of mice. *, P < 0.05; **, P < 0.01; ***, P <0.001 (ANOVA). Scale bars: 100 μm.

We employed a murine model of bleomycin injury-induced lung fibrosis and administered XI-011 via intratracheal inhalation every other day for 10 days to evaluate its effects on lung fibrosis (Figure 6A). Consistent with our *in vitro* results (Figure 2), the protein level of Mdm4 was reduced following treatment with XI-011 in bleomycin-treated fibrotic mice and the control mice (Figure 6B). Also, XI-011 treatment significantly decreased the expression of Col1a1, FN1, and αSMA, especially in bleomycin-treated groups (group 3 vs. group 4) (Figures 6B and S5A). Confocal IF microscopy showed that the expression of MDM4 and αSMA was reduced by XI-011 treatment in bleomycin-induced fibrotic mouse lungs (Figure 6D).

The immunohistochemical analysis further confirmed the reduction of Mdm4 and αSMA (Figure 6E). We also observed that XI-011 inhibited Col1a1 expression in bleomycin-treated fibrotic lungs. Masson's trichrome and HE staining demonstrated fibrotic changes in bleomycin-treated mouse lungs (Figure S5). However, the number of collagen-positive cells (blue) was significantly reduced, and lung morphology was improved in XI-011-treated fibrotic mice compared with vehicle-treated controls (Figure S5). Hyp content assays showed 144 ± 5 μg of Hyp in control mouse lungs (group 3#), which was decreased to 108 ± 9 μg upon treatment with XI-011 (group 4#) (Figure 6C). These data indicated that XI-011 treatment alleviates lung fibrosis in mice.

### XI-011 activates the Mdm4-p53 pathway and promotes clearance of lung myofibroblasts by macrophages in fibrotic mouse lungs

**Figure 7:**
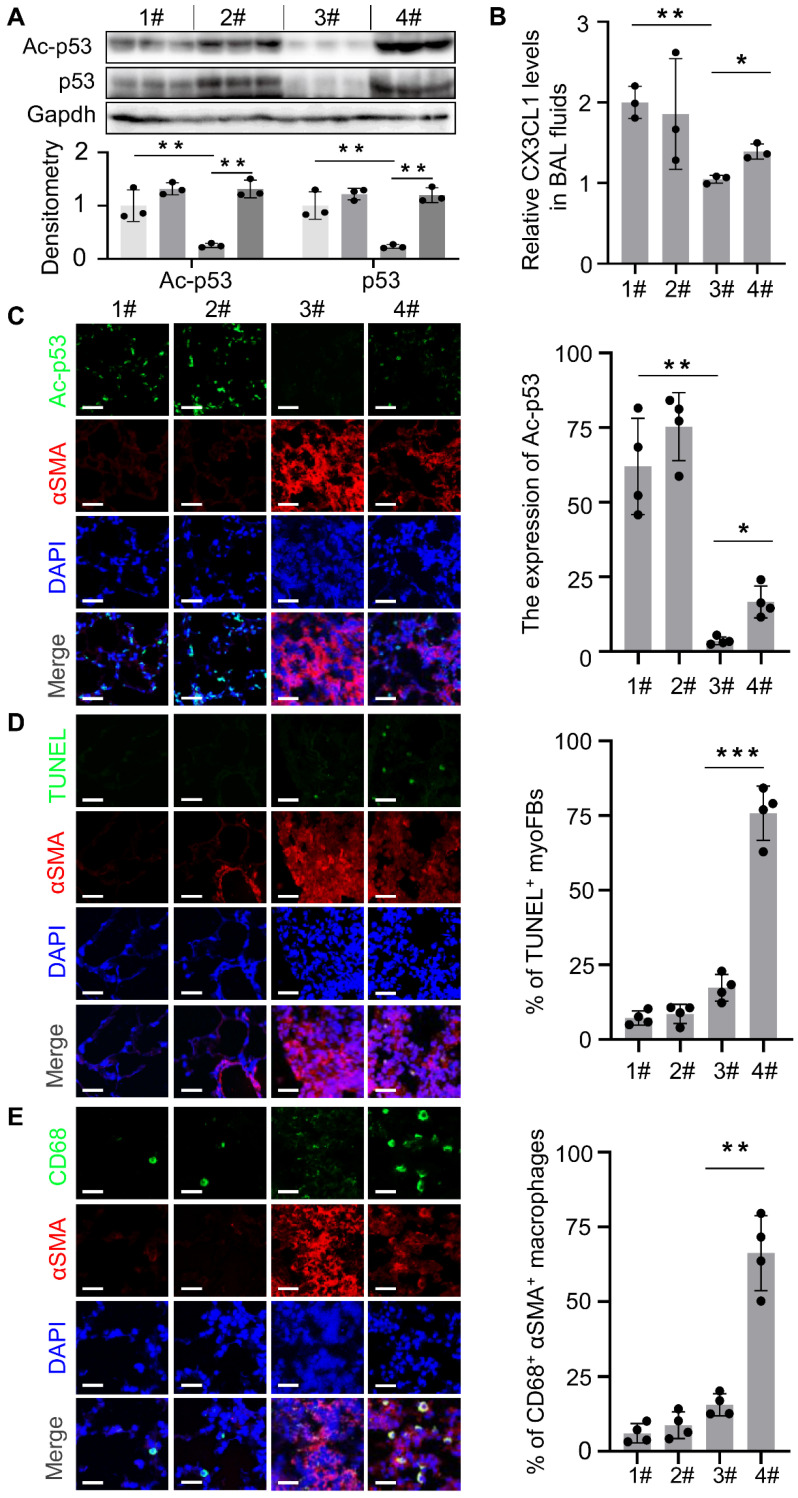
XI-011 activates the MDM4-p53 pathway and promotes the clearance of lung myofibroblasts by macrophages in the fibrotic lungs of mice. (A) Primary lung myofibroblasts were isolated from each group as shown in Figure 6A and the expressions of p53, and Ac-p53 were determined by WB. (B) Levels of CX3CL1 in the BAL fluids of mice in 4 groups were quantified by ELISA (n = 3 per group). (C) Confocal IF staining to evaluate the expressions of Ac-p53 and αSMA in the lung tissues of mice. (D) TUNEL and confocal IF staining to evaluate the apoptosis in the lung tissues of mice. (E) Confocal IF staining to evaluate the expressions of CD68 and αSMA in the lung tissues of mice. Quantitative IF analysis was performed in 4 randomly selected lung areas. Data were presented as the mean ± SD of five mice per treated group. Nuclei were stained with DAPI. *, P < 0.05; **, P < 0.01; ***, P <0.001 (ANOVA). Scale bars: 100 μm.

We previously reported that genetic depletion of Mdm4 in myofibroblasts activated the p53-mediated fibrosis resolution pathway in mice. In the current study, we isolated myofibroblasts from lung tissues in the four groups as indicated in Figure 6A to determine if XI-011 activated p53 in mice. We found that the protein levels of total p53 and acetylated p53 were elevated in normal fibroblasts (group 1# and group 2#) and myofibroblasts (group 3# and group 4#) isolated from mice treated with XI-011 (Figure 7A). Confocal IF assays demonstrated acetylated p53 positive signals in the nuclei of lung myofibroblasts that were treated with XI-011 compared to the vehicle control (Figure 7C).

We previously showed that reducing the expression of Mdm4 promoted lung fibrosis resolution through Fas-mediated apoptosis of lung myofibroblasts and upregulation of DD1α and CX3CL1 promoted the clearance of lung myofibroblasts by macrophages 12. To verify if XI-011 could reverse MDM4 effects in lung fibrosis, we harvested normal and fibrotic mouse lung tissues treated with or without XI-011. Similar to the *in vitro* findings (see Figure 3), XI-011-treated fibrotic lungs displayed more TUNEL-positive nuclei in αSMA-expressing myofibroblasts suggestive of apoptosis (group 3# vs group 4#) (Figure 7D). In contrast, few apoptotic fibroblasts were observed in the controls (group 1# vs group 2#) (Figure 7D). Moreover, we observed more CD68-positive macrophages in XI-011-treated fibrotic lungs compared with the controls (group 3# vs group 4#) (Figure 7E). Importantly, the CD68-positive macrophages were found within αSMA-positive fibrotic lesions, indicating that lung myofibroblasts were engulfed by macrophages (group 4#) (Figure 7E). XI-011 treatment also increased CX3CL1 level in the bronchoalveolar lavage (BAL) fluids of fibrotic lungs, as detected by ELISA (group 3# vs group 4#) (Figure 7B). In contrast, the CX3CL1 level in BAL fluids of normal lungs was not affected (group 1# vs group 2#) (Figure 7B). These results indicated that XI-011 activates the Mdm4-p53 pathway and promotes the clearance of lung myofibroblasts by macrophages in fibrotic lungs in mice.

## Discussion

MDM4 was previously reported as a promising target for IPF treatment. However, a druggable inhibitor of MDM4 for anti-fibrotic therapy was not identified. The inhibitor XI-011 was first recognized as a potent p53 activator by performing a high-throughput screening 20. Later, it was reported that XI-011 activated p53 by inhibiting MDM4 expression 26. The anti-tumor property of this small molecular inhibitor XI-011 was evaluated to block the oncogenic activity of MDM4 in multiple models including *in vitro* cancer cell lines and tumor xenografts in mice 24, 25. The data consistently showed that XI-011 inhibited tumorigenesis via the MDM4-p53-dependent pathway.

MDM4 was discovered as a p53-binding protein and showed a high structural similarity to MDM2 28. While MDM2 E3-ligase activity is known to mono-ubiquitinate p53 protein 29, degradation of p53 requires heterodimer formation between MDM2 and MDM4 30, 31. We previously showed that reducing the expression of MDM4 in lung myofibroblasts increased acetylated p53 level, but total p53 was not affected 12. In this study, lung cells treated with XI-011 showed significant induction of total p53 (Figure 4). One possible explanation for this discrepancy is that the *in vitro* induction of total p53 by knockdown of MDM4 is less sensitive to the stiffness-tunable polyacrylamide gel system, particularly under stiff matrix conditions. Therefore, it seems plausible that XI-011 promotes total p53 expression by directly inhibiting MDM4 expression, thus disabling MDM2/MDM4 dimer formation and, consequently, degradation of p53. Alternatively, XI-011 might activate p53 (Ace-p53), disabling its negative regulator activity for p53-mRNA 32.

A major challenge that impedes the application of potential therapeutics for IPF is their high toxicity. XI-011 showed mild genotoxicity when incubated with T22 cells, even at a high concentration of 25 μM 20. While intraperitoneal XI-011 injection inhibited the growth of xenografted HeLa tumors in mice, the body weight was unaffected by the inhibitor suggesting no apparent toxicity *in vivo* 24. In this study, to minimize toxicity and enhance the anti-fibrosis efficiency of the inhibitor, we delivered XI-011 into the lungs through intratracheal inhalation, a method that enables precise dosing of spontaneously breathing rodents 33. While XI-011 inhibited the proliferation of primary fibroblasts *in vitro* (Figure S3), the *in vivo* administration of XI-011 had only minor effects on the expression of Col1a1 and αSMA (Figure 6D-E). In addition, the Hyp concentration between group 1# and group 2# was comparable (Figure 6C). Significantly, the morphology of normal lungs in mice was not affected by XI-011 treatment (Figure S5B), suggesting the low toxicity of this inhibitor for the treatment of lung fibrosis. These observations further support our conclusion that XI-011 is a promising drug candidate for treating lung fibrosis. Since it remains unclear whether this inhibitor has any side effects on other organs, in the future, comprehensive preclinical studies are required to establish the toxicological profile of the inhibitor.

## Supplementary Material

Supplementary figures.
