# Supplementary material for: Pharmacological inhibition of MDM4 alleviates pulmonary fibrosis
Source: Theranostics. 2023 May 8;13(9):2787–99. doi: 10.7150/thno.81993 (PMC10240813; doi:10.7150/thno.81993)
Supplement: Supplementary file 1 — Supplementary figures. [file thnov13p2787s1.pdf]

Pharmacological inhibition of MDM4 alleviates pulmonary fibrosis

Qianru Mei [1,#], Zhenhua Yang[1,#], Zhengkai Xiang [2,#], He Zuo[1], Zijing Zhou[3], Xiaochuan Dong[4], Ludan Zhang[1], Wenhui Song[1], Yi Wang[1], Qinghua Hu[1], Yong Zhou[5], and Jing Qu[1,*].

Figure legends

Supplemental Figure 1. Representative images show H&E staining and Masson staining of paraffin-embedded lung sections harvested from human normal lung tissue and IPF patient, respectively. Scale bars: 200 μm.

Supplemental Figure 2. Evaluation of the effect of NSC149109 (XI-011) (A) and SJ-172550 (B) on the expressions of indicated items in A549 cells. *, $P < 0.05$; **, $P < 0.01$; ***, $P <0.001$ (ANOVA).

Supplemental Figure 3. CCK8 assay to evaluate the effect of different treatments on cell proliferation. Human (A) or mouse (B) primary lung fibroblasts were isolated and treated with bleomycin/vehicle (PBS) in the presence of XI-011 or DMSO. Myofibroblasts isolated from human IPF (C) or mouse (D) fibrotic lung tissues were treated with XI-011/DMSO in vitro. The absorbance increase at 450 nm was detected and presented as relative values.

Supplemental Figure 4. In vitro treatment of XI-011 has little effect on the apoptosis of epithelial cell. (A) TUNEL and confocal IF microscopy were used to evaluate the effect of XI-011 in the apoptosis of BEAS-2B (A) and MLE12 cells (B). Quantitative IF analysis was performed in 4 randomly selected areas. ***, $P <0.001$ (ANOVA). Scale bars: 100 μm. CCK8 assay to evaluate the effect of different treatments on cell proliferation. BEAS-2B (C) or MLE12 cells (D) were treated with bleomycin/PBS in the presence of XI-011 or DMSO. The absorbance increase at 450 nm was detected and presented as relative values.

Supplemental Figure 5. XI-011 promotes lung fibrosis resolution in mice. (A) Relative mRNA levels of indicated genes in the mice lung tissues. (B) H&E staining and Masson staining of mice lung tissues. *, $P < 0.05$; **, $P < 0.01$; ***, $P <0.001$ (ANOVA). Scale bars: 100 μm.

Figure S1

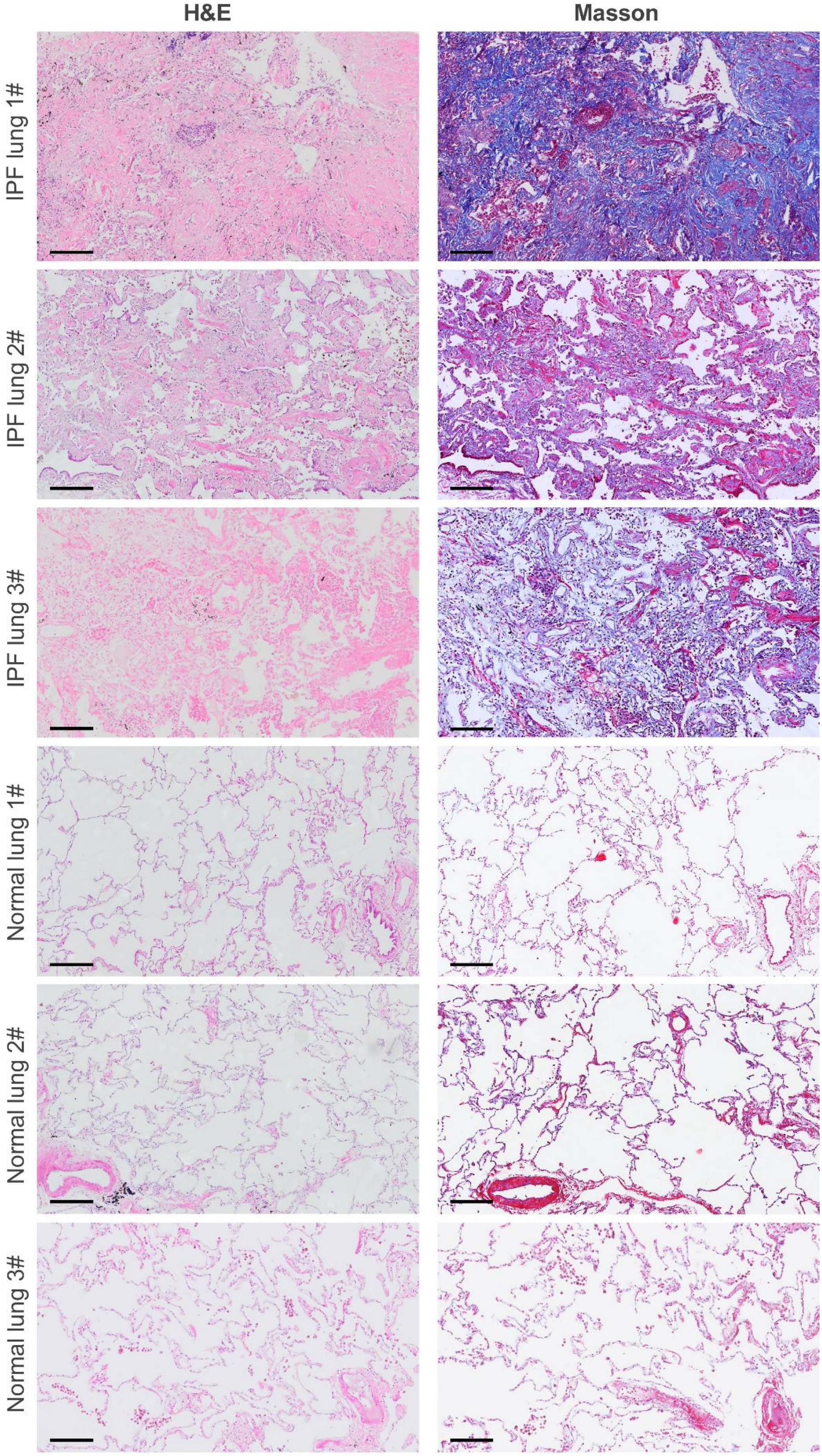
H&E
Masson
IPF lung 1#
IPF lung 2#
IPF lung 3#
Normal lung 1#
Normal lung 2#
Normal lung 3#

Figure S2

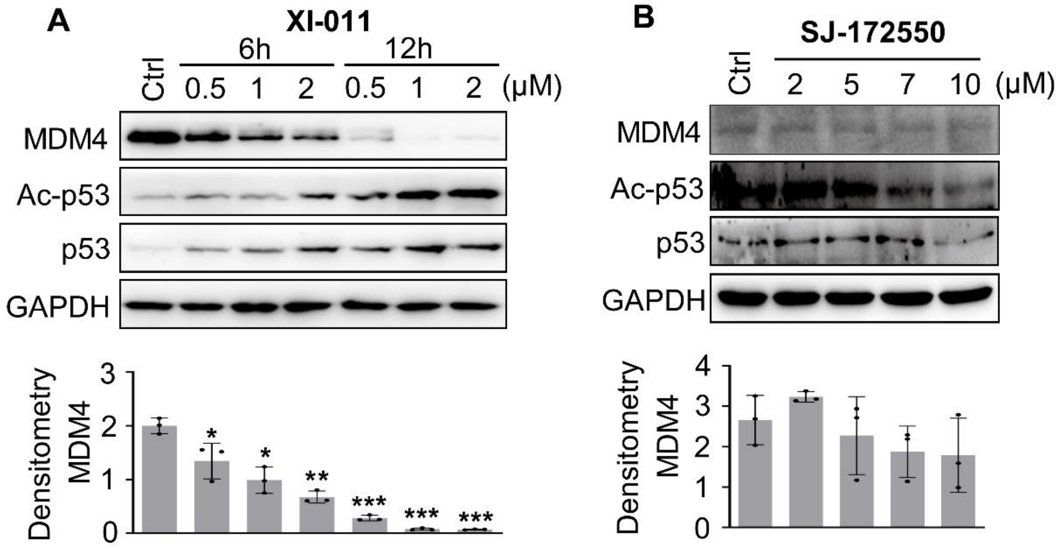

A
XI-011
6h
12h
Ctrl
0.5
1
2
0.5
1
2
(μM)
MDM4
Ac-p53
p53
GAPDH
Densitometry MDM4
3
2
1
0
*
*
**
***
***
***
B
SJ-172550
Ctrl
2
5
7
10
(μM)
MDM4
Ac-p53
p53
GAPDH
Densitometry MDM4
4
3
2
1
0

Figure S3

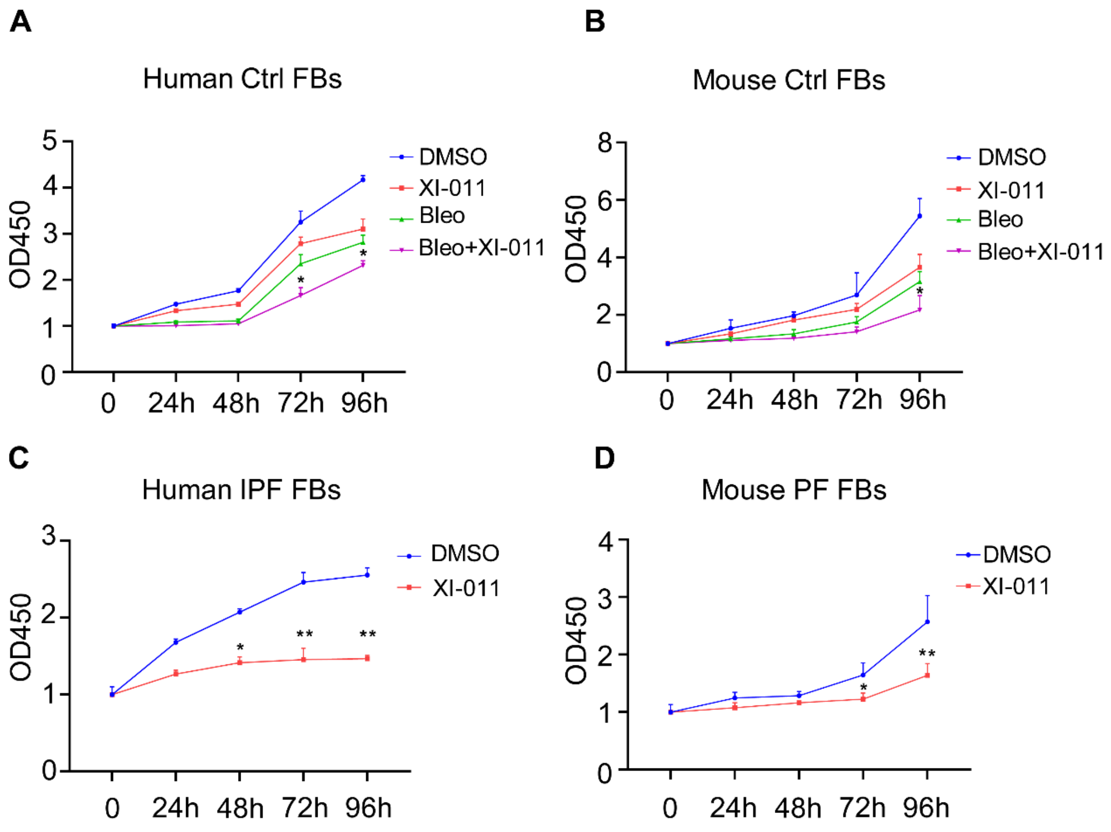
A
Human Ctrl FBs
OD450
DMSO
XI-011
Bleo
Bleo+XI-011
0
24h
48h
72h
96h
B
Mouse Ctrl FBs
OD450
DMSO
XI-011
Bleo
Bleo+XI-011
0
24h
48h
72h
96h
C
Human IPF FBs
OD450
DMSO
XI-011
0
24h
48h
72h
96h
D
Mouse PF FBs
OD450
DMSO
XI-011
0
24h
48h
72h
96h

Figure S4

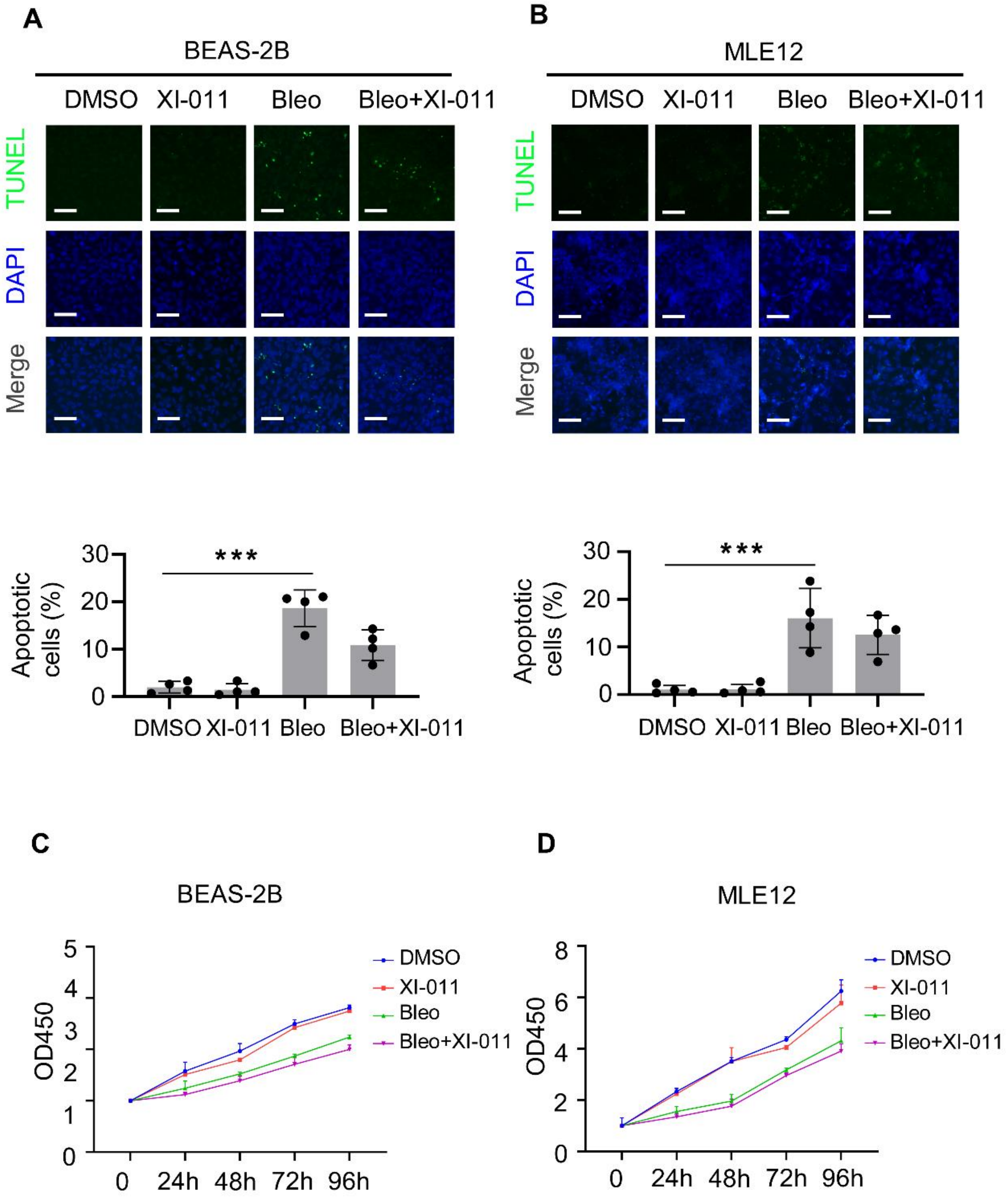
A
BEAS-2B
DMSO
XI-011
Bleo
Bleo+XI-011
TUNEL
DAPI
Merge
B
MLE12
DMSO
XI-011
Bleo
Bleo+XI-011
TUNEL
DAPI
Merge
Apoptotic cells (%)
***
0
10
20
30
DMSO
XI-011
Bleo
Bleo+XI-011
Apoptotic cells (%)
***
0
10
20
30
DMSO
XI-011
Bleo
Bleo+XI-011
C
BEAS-2B
OD450
0
1
2
3
4
5
0
24h
48h
72h
96h
DMSO
XI-011
Bleo
Bleo+XI-011
D
MLE12
OD450
0
2
4
6
8
0
24h
48h
72h
96h
DMSO
XI-011
Bleo
Bleo+XI-011

Figure S5

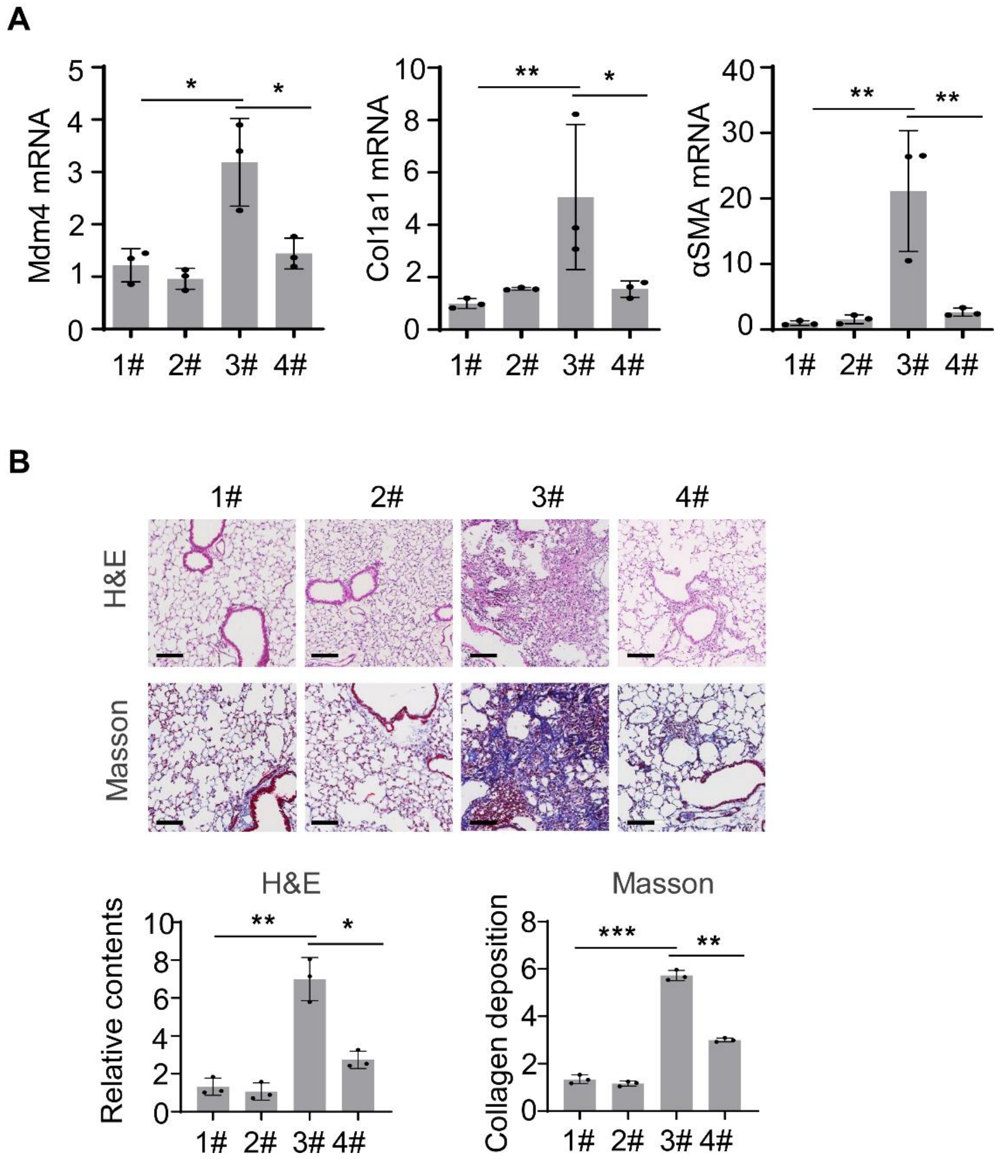
A
Mdm4 mRNA
Col1a1 mRNA
αSMA mRNA
1# 2# 3# 4#
*
**
B
1# 2# 3# 4#
H&E
Masson
Relative contents
Collagen deposition
***
